# Outcomes of mini-monovision with monofocal, enhanced monofocal and extended depth-of-focus intraocular lenses

**DOI:** 10.3389/fmed.2025.1522383

**Published:** 2025-02-21

**Authors:** Issac Levy, Rachana Prashant Shah, Ritika Mukhija, Mayank A. Nanavaty

**Affiliations:** ^1^Sussex Eye Hospital, University Hospitals Sussex NHS Foundation Trust, Brighton, United Kingdom; ^2^Department of Ophthalmology, Rabin Medical Center, Petach Tikva, Israel; ^3^Faculty of Medicine, Tel Aviv University, Tel Aviv, Israel; ^4^Brighton and Sussex Medical School, University of Sussex, Brighton, United Kingdom

**Keywords:** mini-monovision, enhanced monofocal intraocular lenses, extended depth of focus intraocular lens, monofocal intraocular lens, intermediate visual acuity

## Abstract

**Purpose:**

Mini-monovision is a vision correction technique that allows for a broader spectrum of spectacle independence while minimizing anisometropia. This systemic review aims to evaluate the clinical outcomes of pseudophakic mini-monovision with three types of intraocular lenses (IOLs): monofocal, enhanced monofocal, and extended depth of focus (EDOF).

**Methods:**

A comprehensive literature search was conducted using PubMed and MEDLINE to identify studies reporting mini-monovision outcomes within the three categories of IOLs up to July 2024. Inclusion criteria were studies with more than 20 patients, target refraction to achieve mini-monovision difference in the fellow eye, and minimum follow-up of 3 months. The primary outcome measure was uncorrected binocular intermediate visual acuity (UCIVA). The secondary outcomes were binocular uncorrected distance visual acuity (UCDVA), binocular uncorrected near visual acuity (UCNVA), patient-reported outcomes measures (PROMs), spectacle independence, contrast sensitivity, photic phenomenon, enhancement surgeries and IOL exchange.

**Results:**

A total of 113 studies were screened, of which 19, with a total of 1,530 patients, were eligible for inclusion in this review. Mean logMAR binocular UCIVA was 0.16 ± 0.01, 0.11 ± 0.06, 0.08 ± 0.07 (*p* = 0.41), and mean logMAR UCDVA was 0.08 ± 0.05, 0.04 ± 07, 0.04 ± 0.04 (*p* = 0.36), in the monofocal, enhanced monofocal, and EDOF groups, respectively. The mean spectacle independence rate was 51% ± 22.1, 55% ± 35.4 and 63.4% ± 24.6 (*p* = 0.05), respectively, in the monofocal, enhanced monofocal and EDOF groups. A comparable low incidence of halos and glare was observed when enhanced monofocal lenses were evaluated against traditional monofocal lenses. EDOF lenses have, however, demonstrated mixed results. The complications, IOL exchange, and excimer laser enhancement rates were low across all groups.

**Conclusion:**

While enhanced monofocal and EDOF IOLs may provide slightly better binocular intermediate visual outcomes and higher spectacle independence compared to monofocal lenses with regards to mini-monovision and intermediate vision performance, the differences are not statistically significant. All three IOL types exhibit high patient satisfaction rates when choosing a mini-monovision approach with decreased dependence on spectacles.

## Introduction

In the ever-evolving landscape of cataract surgery, the scales have now tilted more toward providing refractive correction rather than its original purpose of visual rehabilitation. Advances and innovations in technology have significantly improved the surgical management of presbyopia (1). Given the change in visual needs over time, patients’ expectations for excellent visual performance and spectacle independence not only for distant vision but also for intermediate and near, mainly due to daily tasks that require this range of vision (tablet and smartphone reading, working on computers, driving), have substantially increased (2). Current armamentarium of pseudophakic presbyopia corrections for cataract surgeons primarily include (1) implantation of multifocal intraocular lenses (IOLs), (2) implantation of extended-depth of focus (EDOF) IOLs (3), (3) implantation of accommodative IOLs, and (4) pseudophakic monovision with monofocal IOLs (4).

Multifocal IOLs usually provide high rates of spectacle independence; however, they could be associated with visually significant photic phenomena due to light distribution into multiple foci, especially if patient selection is inappropriate (5–7). Traditional monovision with monofocal IOLs, wherein the dominant eye is targeted for distance emmetropia and the non-dominant is targeted for a near emmetropia leaving a residual myopic error, has been used to overcome the photic phenomena of multifocal IOLs. More recently, mini-monovision with monofocal IOLs, wherein the non-dominant eye is targeted for a relatively smaller residual myopia of −0.75 D to −1.50 D, has been employed and has achieved similar results. This technique also helps in reducing to a greater extent the rate of positive dysphotopsias, being harmless for stereopsis compared to traditional monovision (8). When the non-dominant eye is chosen for distance vision, the technique is crossed monovision. The prevalence of monovision or mini-monovision after cataract surgery is rarely reported in the literature and varies according to clinical practices and the studied population, ranging from 22 to 34% (9). The prevalence can depend on factors such as patient preference, surgeon recommendation, and pre-surgical considerations like the patient’s tolerance to anisometropia (9).

In the hybrid monovision technique, a diffractive multifocal IOL is implanted in the non-dominant eye, whereas a monofocal IOL is implanted in the dominant eye (2). The most widely used approach is the implantation of monofocal IOL in both eyes because of the relatively low-cost of monofocal lenses and satisfying performances for far vision restoration (8, 10).

Extended depth of focus IOLs create an elongated focal point to extend the range of vision and decrease photic phenomena by eliminating overlapping far and near images, thereby accepting some compromise for near-vision (3, 11, 12). The mini-monovision approach has also been successfully adopted with EDOF IOLs (13–20). Another strategy is to employ so-called enhanced monofocal IOLs. These IOLs possess a high-order aspheric anterior surface with a continuous change in power from the periphery toward the lens center (21). This characteristic creates a modified anterior surface with a small central zone designed to extend the depth of focus and consequently improve intermediate vision while maintaining good performance at a distance (22–24) and higher patient satisfaction than classic monofocal IOL (25–28). This maintains a profile similar to photic phenomena in aspheric monofocal IOLs (28). Mini-monovision with these enhanced monofocal IOLs also improves patient satisfaction with low dysphotopsia (21, 29, 30).

The primary objective of this study was to review published literature regarding the efficacy of pseudophakic mini-monovision using monofocal, enhanced monofocal and EDOF IOLs in the correction of presbyopia after cataract extraction in comparison to each other, based on objective parameters, including visual acuity (VA) at near, intermediate and distance and possible complications postoperatively and subjective parameters like patient satisfaction and spectacle independence.

## Materials and methods

A comprehensive literature search was conducted to identify studies reporting mini-monovision outcomes after cataract surgery. We included studies that specify mini-monovision refraction targeting in their abstract. We compared three different intra-ocular lens types: monofocal, enhanced monofocal and EDOF IOLs. Inclusion criteria were retrospective or prospective studies published until August 2024 with a minimum of 20 patients and a minimum follow-up of 3 months published in peer-reviewed journals. Studies not published in the English language were excluded.

A systematic literature search for related studies was carried out on PubMed and MEDLINE using the Medical Subject Headings (MeSH) terms “mini-monovision,” “monovision,” “monofocal,” “pseudophakic mini-monovision,” “enhanced monofocal,” “EDOF” and “extended depth of focus.” The Boolean operators “AND” and “OR” combined these MeSH terms and search studies on mini monovision with either of the three IOLs. The initial search was performed without any filters or language restrictions. Data published in any other language but English was excluded from the study. The titles and abstracts resulting from the searches were reviewed. A full-text copy of all potentially relevant studies was reviewed for eligibility, and only those studying mini monovision using monofocal, enhanced monofocal or EDOF IOLs were included in the study.

The risk of bias in the articles was assessed using the RoB version 2 tool which considered the following factors: Random sequence generation and allocation concealment (selection bias), blinding of participants (performance bias), blinding of outcome assessment (detection bias), missing outcome data (attrition bias) and selective reporting (reporting bias).

Data collection was performed on an Excel 365 spreadsheet (Microsoft, Redmond, WA, United States) outlining all the relevant parameters. The primary outcome measure was uncorrected binocular intermediate visual acuity (UCIVA). The secondary outcomes were binocular uncorrected distance visual acuity (UCDVA), binocular uncorrected near visual acuity (UCNVA), patient-reported outcomes measures (PROMs), spectacle independence, contrast sensitivity, photic phenomenon, enhancement surgeries and IOL exchange. One review author inputted the data into the spreadsheets; another author re-checked and validated it. Any disagreements regarding the inclusion or exclusion of the studies were resolved through discussion among the authors. We used the data from the latest follow-up visit. The Kolmogorov–Smirnov test was performed to assess the normality of the data distribution, and it was found to have a normal distribution. Then, statistical analysis was performed using Single-factor ANOVA to assess the differences across groups. The level of statistical significance is set at *P* < 0.05. All visual acuity data were standardized by converting them to logMAR format when originally presented in Snellen or decimal formats. This conversion allowed for uniformity in the measurement scale, enabling more precise statistical interpretation and comparison across datasets. Of all the studies in the three different categories, only Sevik et al. (16) and Lee (20) from the EDOF group reported statistical powers of 0.8 and 0.9, respectively.

## Results

**FIGURE 1:**
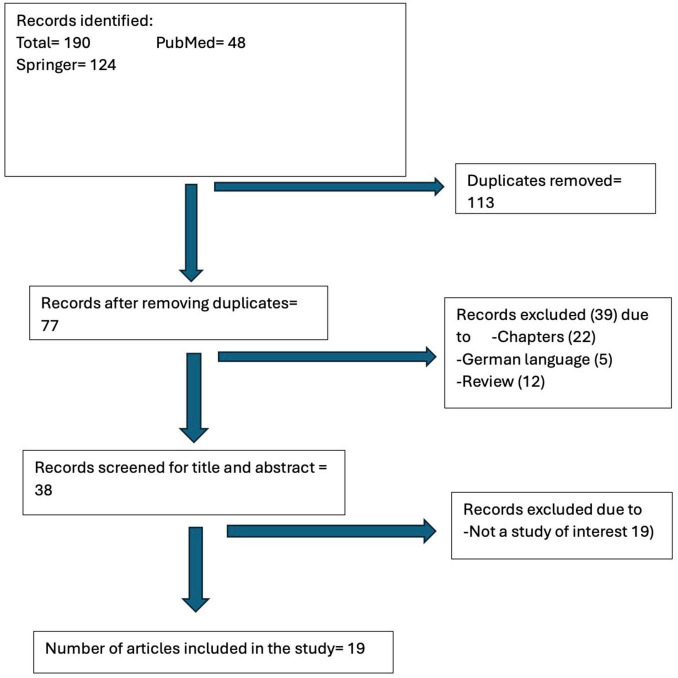
Study flowchart.

This review included a total of 19 studies published over 17 years involving 1,530 patients (Figure 1). A total of one German article, four German Conference abstracts, 22 chapters and 12 Review articles were excluded (Supplementary Table 1). There were seven studies within the monofocal group (31–37) four in the enhanced monofocal group (22, 38–40), and eight in the EDOF group (13–16, 20, 41–43). The included studies are summarized in Table 1 and the overall demographics in Table 2.

**TABLE 1:** All included studies.

	Study	Number of patients	Mean age	Gender (Female%)	F/U period (months)	Refractive aim in the non-dominant eye	Binocular UIVA (LogMar)	Binocular UDVA (LogMar)	Binocular UNVA (LogMar)	Spectacle independence (%)	Limitations
**Monofocal lens**											
1	Wróbel-Dudziñska et al. (31)	463	63.5 ± 11.29	48	120	−0.75 to −1.5	NA	0.16 ± 0.20	0.05 ± 0.1	72	Retrospective, non-comparative.
2	Goldberg et al. (32)	56	67.5	44	6	−1.25 to −1.50	NA	0.09 ± 0.09	0.15 ± 0.14	73	Limited number of activities assessed in the questionnaire, and the lack of comparison data on patient satisfaction outcomes with other options.
3	Hafez and Helaly (37)	30	56.3 ± 5.5	33.00	3	−1 to −1.5	0.17 ± 0.14	0.09 ± 0.07	0.31 ± 0.19	43	Retrospective, small sample size, lack of comparative group, short follow-up.
4	Chen et al. (33)	20	NA	50	3	−0.50 to −1.25	NA	Better than 0.2 in all the patients	Better than 0.3 in all the patients	35	Retrospective, small sample size, short follow-up.
5	Zhang et al. (34)	22	67	68.18	3	−2.00~	0.3 or better in 73%	0.1 or better in 86%	0.1 or better in 91%	77	Retrospective, small sample size, short follow-up.
6	Wilkins et al. (35)	73	68.7 ± 12	57.5	4	−1 to −1.5	0.15 ± 0.12	0.06 ±0.16	0.01 ±0.12	25.8	Short follow-up
7	Labiris et al. (36)	38	59.5 ± 10.4	52	6	−1.25	NA	0.02	0.14	31.4	Small sample size.
**Enhanced monofocal lens**											
1	Park et al. (22)	25	71.92 ± 9.98	−52	3	−0.75	0.12 ± 0.09	0.1 ± 0.11	0.06 ± 0.06	80	Short follow-up, small sample size., Short follow-up
2	Beltraminelli et al. (38)	37	73.24 ± 1.13	51.35	3	−0.50 to −1.25	0.1	0.1	0.35	NA	Retrospective, small sample size, short follow-up.
3	Dell et al. (39)	383	60.07 ± 8.37	47.8	1	= −0.50 D	0.18 ± 0.18	−0.03 ± 0.1	0.42 ± 0.19	Overall satisfaction 93.2	Retrospective, short follow-up.
4	Sandoval et al. (40)	37	70 ± 4	37.84	3	−0.75 D	0.04	0	0.21	30	Retrospective, small sample size, short follow-up.
**EDOF lens**											
1	Won et al. (13)	27	63.19 ± 3.95	59.3	3	−0.1 to −0.5	−0.03 ± 0.07	0.07 ± 0.1	0.22 ± 0.12	NA	Retrospective, small sample size, short follow-up.
2	Campos (14)	20	65.00	50	3	−0.50	0.18 ± 0.16	0.09 ± 0.25	0.32 ± 0.10	65.00	Retrospective, small sample size, short follow-up.
3	Tomagova et al. (41)	62	70.60	44	3	−0.50	0.13 ± 0.11	−0.02 ± 0.07	0.4 ± 0.20	34.00	Retrospective, non-comparative, short follow-up
4	Kim et al. (42)	61	61.80	59	24	–0.60	0.056 ± 0.04	0.086 ± 0.09	0.14 ± 0.05	85.00	Retrospective, non-comparative
5	Sevik et al. (16)	14	63.43	42.86	6	−0.50	−0.03 ± 0.07	−0.03 ± 0.05	0.15 ± 0.07	85.70	Small sample size
6	Fernandes et al. (15)	63	68.50	74	3	−0.75	NA	0.05 ± 0.01	0.16 ± 0.03	75.00	Short follow-up
		66	68.00	68	3	−0.75	NA	0.06 ± 0.01	0.21 ± 0.02	75.00	
7	Vasavada et al. (43)	33	60.96	45.7	6	= 0.5 to = 0.75 D	0.07 ± 0.08	0.028 ± 0.05	0.22 ± 0.03	24.20	Small sample size
8	Lee (20)	30	61.60	53	3	−0.50	0.1 ±0.1	0.06 ± 0.12	0.27 ± 0.10	NA	Retrospective, small sample size, non-comparative, short follow-up.

F/U, follow up; UCIVA, uncorrected intermediate visual acuity; UCDVA, uncorrected distance visual acuity; UCNVA, uncorrected near visual acuity; EDOF, extended depth of focus.

**TABLE 2:** Overall demographics.

IOL type	Number of studies	Follow-up period (months)	Number of patients	Age (years)	Gender (female %)
Monofocal	7 (30–36)	20.7 ± 43.8	100.3 ±63.8	63.7 ± 5	40.8 ± 22.3
Enhanced monofocal	4 (21, 3–39)	2.5 ± 1.2	120.5 ± 203.3	68.8 ± 7.3	47.3 ± 2.3
EDOF	8 (13–16, 40–43)	8.4 ± 9.6	44 ± 22.4	64.1 ± 3.7	58.1 ± 15.5

### Primary outcome

In the monofocal group the binocular UCIVA was reported only in three of these studies (33, 35, 36). Two studies found a mean of 0.16 ± 0.01 logMAR (criteria of monovision as target postoperative refraction between −0.75 D and −1 D) (35, 37) and one (34) described 0.3 logMAR or better in 73% of the patients (criteria of mini-monovision as target postoperative refraction approximately −2 D) (Table 1). In the enhanced monofocal group the mean binocular UCIVA was 0.11 ± 0.06 logMAR (criteria of mini-monovision as target postoperative refraction between −0.50 D and −0.75 D) and in the EDOF group the mean binocular UCIVA was 0.08 ± 0.07 logMAR (criteria of mini-monovision as target postoperative refraction in all studies up to −0.75 D), respectively. No statistically significant difference was found between the different intraocular lens types (*p* = 0.41) (Table 1).

### Secondary outcomes

There was no statistically significant difference when comparing mean binocular UDCVA, UCNVA and spectacle independence rates between the different intraocular lens types. The main secondary outcomes are presented in Table 3.

**TABLE 3:** The secondary outcomes.

Outcome	Monofocal	Enhanced monofocal	EDOF	*P*-value
Binocular UCDVA	0.08 ± 0.05 (*n*=5)	0.04 ± 0.07 (*n*=4)	0.04 ± 0.04 (*n*=8)	0.34
Binocular UCNVA	0.13 ± 0.11 (*n*=5)	0.26 ± 0.16 (*n*=4)	0.20 ± 0.11 (*n*=8)	0.36
Spectacle independence	51 ± 22.12% (*n*=7)	55% ± 35.4 (*n*=2)	63.4 ± 24.6% (*n*=6)	0.79
Contrast sensitivity*	1.69 ± 0.4 (*n*=2)	–	1.55 ± 0.03 (*n*=3)	–

*Pellie Robson contrast sensitivity test.

### Patient-reported outcomes measures

#### Spectacle independence

The mini-monovision technique demonstrated that the three types of IOL groups achieved an overall spectacle independence of 50% or more. Patients reported high satisfaction levels, with low rates of needing refractive correction for distance and intermediate vision. Additionally, no statistical differences were observed between the groups (*p* = 0.78). Within the monofocal group, patients reported a high satisfaction rate with high variability of nearly complete spectacle independence from 25 to 77% (31–37). In the enhanced monofocal group–all studies showed a high satisfaction rate ranging from 84 to 96%, (22, 40) with most patients reporting they would recommend the procedure to others (39). EDOF group– patients showed a high satisfaction rate and variable complete spectacle independent rate from 24 to 75% (13–16, 41–43).

#### Quality of vision

Studies comparing contrast sensitivity performance in enhanced monofocal lenses versus traditional monofocal lenses found good performance and no statistically significant difference under low and high luminance conditions for any spatial frequency (22, 38, 40, 44).

In the monofocal group, studies observed minimal to no occurrences of clinically significant photic phenomena. Although some studies reported an absence of halos and glare, it is important to note that these studies did not directly inquire about patients’ experiences (31, 32, 37). As expected, significantly fewer complaints of positive photopic phenomena were found compared to those reported with multifocal lenses (1–3, 34–36). A comparable low incidence of halos and glare was observed when enhanced monofocal lenses were evaluated against traditional monofocal lenses (22, 39, 40, 44). EDOF lenses have, however, demonstrated mixed results. Some studies found a similar rate of positive photopic phenomena as in traditional monofocal lenses (16, 20, 43) whilst others experienced frequent halos and glare (14, 41).

#### Rates of repeat surgical procedures

Intraocular lenses exchange can be offered in cases of patient dissatisfaction due to non-resolved, intolerable, positive dysphotopsias, residual refractive error, or refractive surprise. IOL exchange rate and secondary corneal enhancement therapy were reported only within the monofocal groups. Only two studies provided data on the incidence of secondary corneal enhancement procedures performed via laser vision correction. One study recorded an incidence of 1%, whereas the other reported an incidence of 9.7% (31, 35). Four studies (31, 32, 34, 35) provided data on intraocular lens exchange rate. Goldberg et al. (32) reported an exchange rate of 3.6%, corresponding to two patients, while three other studies indicated no cases of IOL exchange (2–4).

#### Risk of bias

Six randomized controlled trials [Labiris et al. (36), Sandoval et al. (40), Wilkins et al. (35), Sandoval et al. (17), Sevik et al. (16), Vasavada et al. (43)] were assessed for risk of bias using RoB tool version 2 under five domains (Table 4). Four trials employed the method of computerized randomization and were assessed as low risk. The methods of randomization and allocation concealment employed by Sandoval et al. (40) and Sevik et al. (16) have not been specified, and there could be some concerns pertaining to the risk of bias in these trials. Low risk of bias was seen in the rest of the domains for all trials. Assessment of risk of bias using RoB 2 tool could not be performed for the remaining thirteen studies, as they are observational studies.

**TABLE 4:** Risk of bias.

Signaling questions	Labiris et al. (36)	Sandoval et al. (40)	Wilkins et al. (35)	Sevik et al. (17)	Fernandes et al. (16)	Vasavada et al. (43)
**Domain 1: risk of bias arising from the randomization process**						
Was the allocation sequence random?	Y/PY	NI	PY/Y	PY/Y	NI	Y/PY
–	Custom computer randomization program used	Not specified	A minimization program was used	Online integer generator used	Not specified	Computer generated random number tables used
Was the allocation sequence concealed until participants were enrolled and assigned to interventions?	Y/PY	NI	PY/Y	Y/PY	Y/PY	Y/PY
Did baseline differences between intervention groups suggest a problem with the randomization process?	PN/N	PN/N	PN/N	PN/N	PN/N	PN/N
Risk-of-bias judgment	Low	Some concerns	Low	Low	Some concerns	Low
**Domain 2: risk of bias due to deviations from the intended interventions (effect of assignment to intervention)**						
Were participants aware of their assigned intervention during the trial?	NI	PN/N NI	N/PN	Y/PY	PN/N	Y/PY
Were carers and people delivering the interventions aware of participants’ assigned intervention during the trial?	PY/Y		PY/Y	NI	NI	NI
Were there deviations from the intended intervention that arose because of the trial context?	PN/N	PN/N	PN/N	PN/N	PN/N	PN/N
Was an appropriate analysis used to estimate the effect of assignment to intervention?	Y/PY	Y/PY	Y/PY	Y/PY	Y/PY	Y/PY
Risk-of-bias judgment	Low	Low	Low	Low	Low	Low
**Domain 2: risk of bias due to deviations from the intended interventions (effect of adhering to intervention)**						
Were participants aware of their assigned intervention during the trial?	NI	Y/PY NI	Y/PY	Y/PY	Y/PY	Y/PY
Were carers and people delivering the interventions aware of participants’ assigned intervention during the trial?	NI	–	PY	NI	NI	NI
Were important non-protocol interventions balanced across intervention groups?	Y/PY	Y/PY	NI	Y/PY	Y/PY	Y/PY
Were there failures in implementing the intervention that could have affected the outcome?	PN/N	PN/N	PN/N	PN/N	PN/N	PN/N
Was there non-adherence to the assigned intervention regimen that could have affected participants’ outcomes?	PN/N	PN/N	PN/N	PN/N	PN/N	PN/N
Was an appropriate analysis used to estimate the effect of adhering to the intervention?	NI	NI	NI	NI	NI	NI
Risk-of-bias judgment	Low	Low	Low	Low	Low	Low
**Domain 3: missing outcome data**						
Were data for this outcome available for all, or nearly all, participants randomized?	Y/PY	Y/PY	Y/PY	Y/PY	Y/PY	Y/PY
Risk-of-bias judgment	Low	Low	Low	Low	Low	Low
**Domain 4: risk of bias in measurement of the outcome**						
Was the method of measuring the outcome inappropriate?	PN/N	PN/N	PN/N	PN/N	PN/N	PN/N
Could measurement or ascertainment of the outcome have differed between intervention groups?	PN/N	PN/N	PN/N	PN/N	PN/N	PN/N
Were outcome assessors aware of the intervention received by study participants?	Y/PY	Y/PY	PN/N	PN/N	PN/N	PN/N
Could assessment of the outcome have been influenced by knowledge of intervention received?	PN/N	PN/N	PN/N	PN/N	PN/N	PN/N
Risk-of-bias judgment	Low	Low	Low	Low	Low	Low
**Domain 5: risk of bias in selection of the reported result**						
Were the data that produced this result analyzed in accordance with a pre-specified analysis plan that was finalized before unblinded outcome data were available for analysis?	NI	NI	NI	PN/N	PN/N	PN/N
Is the numerical result being assessed likely to have been selected, on the basis of the results, from	–	–	–	–	–	–
…. multiple eligible outcome measurements (e.g., scales, definitions, time points) within the outcome domain?	PN/N	PN/N	PN/N	PN/N	PN/N	PN/N
… multiple eligible analyses of the data?	PN/N	PN/N	PN/N	PN/N	PN/N	PN/N
Risk-of-bias judgment	Low	Low	Low	Low	Low	Low
**Overall risk of bias**						
Risk-of-bias judgment	Low	Some concerns	Low	Low	Some concerns	Low

Y, yes; PY, probably yes, NI, not included; N, no; PN, probably no.

## Discussion

Our review of 19 studies, including 1,530 patients, found that the mini-monovision technique in cataract surgery, whether using traditional monofocal lenses or more advanced options like enhanced monofocal or EDOF lenses, can be an effective alternative for patients seeking glasses independence (Table 1). The definition of mini-monovision targeting low myopia varies in the literature from a residual refractive error of −0.75 D to −2.00 D in the non-dominant eye (Table 1).

Traditional cataract surgery, involving monofocal lens implantation, significantly improves visual acuity, predominantly for distance vision. However, these lenses offer a limited depth of focus, resulting in a considerable reliance on refractive correction for various daily activities in the intermediate and near vision ranges. The increasing working age and the increased use of computers, tablets, and smartphones as an integral part of almost every daily activity results in decreased functional vision and the need for a cost-effective solution (45). Today, we can offer several types of IOLs to help our patients gain functional vision at a broader range of distances (2), Patients have varying needs and personalities, and some may have ocular comorbidities that can impact their vision. Considering these factors, it’s essential to tailor the choice of intraocular lens to each individual. Multifocal IOLs can provide a wide range of vision; however, they may also lead to an increase in positive dysphotopsias, and a decrease in contrast sensitivity and overall visual quality. Therefore, these lenses are not suitable for everyone (5, 46, 47). As discussed earlier, enhanced monofocal and EDOF IOLs can improve depth-of-focus while providing better intermediate visual acuity and maintaining vision quality similar to monofocal IOLs (48).

We found no statistically significant differences in binocular UCIVA, UCDVA and UCNVA between the three lens types (Table 1). Enhanced monofocal and EDOF lenses, engineered to provide a broader range of vision, did not show significant clinical advantages in this review with regard to UCIVA with mini-monovision (Tables 1, 3). Current literature suggests enhanced monofocal lenses perform slightly better or are comparable to standard monofocal lenses in the distance and intermediate vision (20). In contrast, EDOF lenses are associated with improved intermediate and near visual acuity outcomes (25–30, 49–51).

Patient satisfaction was homogeneously high across all three groups, with most patients reporting positive experience and a decreased need for refractive correction. Spectacle independence rates were reported across all three groups, with rates above 50% in most studies. Enhanced monofocal and EDOF lenses showed slightly incremental improvement, but the difference was not statistically significant. This suggests that monofocal lenses still provide reasonable spectacle independence when using the mini-monovision technique. Although we did not find statistically significant difference in the primary outcomes of UCIVA between the groups, it has to be noted the mean UCIVA was best with EDOF, followed by enhanced monofocal and then monofocal lens (Table 1). Similarly although there was no statistically significant difference in mean percentage of patients achieving spectacle independence (Table 3), spectacle independence was highest in EDOF, followed by enhanced monofocal and then monofocal groups, respectively (Table 3). The lack of statistical significant despite of changes in mean UCIVA and spectacle independence may be attributable to low number included studies in each group, variable definition of mini-monovision used and variations in study designs.

Contrast sensitivity is essential for good functional vision, particularly in low-light settings. It significantly affects visual performance and the ability to distinguish objects and details in those challenging conditions. Contrast sensitivity performance showed no significant differences between traditional monofocal and enhanced monofocal lenses under low and high luminance conditions, confirming that both IOL types offer comparable outcomes. However, EDOF lenses showed some variability, with studies reporting similar or higher halos and glare rates than monofocal lenses (38, 52, 53).

Extended depth of focus lenses are categorized: diffractive, refractive, and hybrid. Halos and glare are particularly associated with diffractive lens designs. Diffractive EDOF IOLs use microstructures to split light into multiple focal points, extending the depth of focus. This can result in scattering, creating positive visual aberrations like halos, especially in low lights. Refractive EDOF IOLs are generally less prone to these effects but can still cause halos and glare if their refractive zones lead to variations in light entering the eye. Overall, these artefacts arise from the lens’s attempt to provide extended vision ranges, which can lead to imperfections in light processing (3, 12, 54). This suggests a need for caution when recommending EDOF lenses to patients sensitive to photic phenomena. Interestingly, enhanced monofocal lenses displayed a low incidence of halos and glare, supporting their value for patients desiring minimal photic disturbances (22, 39, 40, 44). Our review raises an important question–should we offer mini-monovision to all patients undergoing cataract surgery? Patients need to be assessed thoroughly before surgery to understand their needs and functional vision requirements. One way to provide this insight is to use the validated questionnaires in addition to open conversation (55). According to the patient’s ocular history and vision needs, the suitable type of IOL can be selected with the appropriate refractive target. The mini-monovision approach can be particularly beneficial for patients with a high priority on distance vision but requiring only functional intermediate vision. Mini-monovision may provide a reasonable solution by incorporating mild anisometropia. Such a tailored approach, including enhanced monofocal IOLs, should be considered standard practice in cases where full presbyopia correction is either not possible, or not deemed necessary or desired, thereby helping patients achieve greater satisfaction in both visual function and vision-related quality of life.

High levels of safety were found with all lens types using the mini-monovision technique. We describe low rates of IOL exchange and secondary corneal enhancement procedures. IOL exchange was rarely done, which on sight contrast to multifocal IOLs, which are associated with higher rates of IOL exchange due to dissatisfaction with visual quality or photic phenomena. Only two studies provided data on the incidence of secondary corneal enhancement procedures performed via laser vision correction. One study recorded an incidence of 1%, whereas the other reported an incidence of 9.7% (31, 35). Four studies (31, 32, 34, 35) provided data on intraocular lens exchange rate. Goldberg et al. (32) reported an exchange rate of 3.6%, corresponding to two patients, while three other studies indicated no cases of IOL exchange (2–4). There are different reasons for IOL exchange, Goemaere et al. (56) reported a 15 years studies regarding IOL exchange and found IOL opacification to be the primary reason (28%), with multifocal IOL being second with 15%. Dissatisfaction with multifocal IOL can be effectively addressed, not only by exchanging to monofocal IOL but also by selecting an alternative multifocal IOL design (57, 58). Laser vision correction as a secondary enhancement procedure was reported only in two studies (31, 35), with incidences ranging from 1 to 9.7%, suggesting that while secondary interventions are rare, but sometimes necessary to optimize visual outcomes in patients with residual refractive errors (59).

The definition of mini-monovision remains ambiguous, with no clear consensus on the precise refractive targets or optimal range of anisometropia. Traditionally, monovision involves correcting one eye for distance vision and the other for near (60, 61) but mini-monovision aims for a smaller interocular difference, providing a broader range of functional vision with minimal discomfort. In this review, most studies in the monofocal group targeted the myopic eye from −0.75 D up to −1.5 D and one even −2.00 D, (31–37) as opposed to the enhanced monofocal and EDOF lens, where the target was up to −0.75 D (13–16, 20, 41–43). This course can be explained by the broader range of focus these lenses provide. While the ideal target may vary between patients, this range offers a practical compromise for functional vision across distances.

The limitations included an unequal number of studies in each group and variations in study design; some studies had two arms and differing follow-up durations, which may have affected the outcomes. Additionally, not all parameters were reported consistently across the studies, and there is no standard definition of mini-monovision. Furthermore, the outcomes of the enhanced monofocal and EDOF IOLs may vary based on their refractive or diffractive optical designs too and this review does not differentiate the IOLs based on their refractive or diffractive designs. Pertaining to the risk of bias in the trails, two studies did not mention the method of randomization employed. This could raise some concerns regarding risk of bias in these trials. No robust studies comparing two groups of lenses directly for mini-monovision outcomes were identified. Moreover, not all included studies report the statistical power. Further studies are request comparing these three groups of IOLs with adequate statistical power to ensure statistical significance for UCIVA and spectacle independence with mini-monovision.

In summary, our review suggests that, whilst enhanced monofocal and EDOF IOLs may provide slightly better intermediate visual outcomes and higher spectacle independence compared to monofocal lenses with regards to mini-monovision and intermediate vision performance, the differences are not statistically significant. All three IOL types exhibit high patient satisfaction rates when choosing a mini-monovision approach with decreased dependence on spectacles. Monofocal and enhanced monofocal showed the lowest incidence of positive dysphotopsia and comparable contrast sensitivity performance. These findings support using all three lens types depending on patient preferences. Future studies should focus on long-term outcomes and employ standardized tools for evaluating visual performance and patient satisfaction.
