# Supplementary material for: Outcomes of mini-monovision with monofocal, enhanced monofocal and extended depth-of-focus intraocular lenses
Source: Front Med (Lausanne). 2025 Feb 21;12:1522383. doi: 10.3389/fmed.2025.1522383 (PMC11885283; doi:10.3389/fmed.2025.1522383)
Supplement: Supplementary file 1 [file Table_1.docx]

Supplementary Table 1. Excluded articles.

**Articles in German language**

1. Hienert, J., Findl, O. IOLs mit erweiterter Tiefenschärfe. *Spektrum Augenheilkd.* **33**, 169–171 (2019). <https://doi.org/10.1007/s00717-019-00441-6>

**Conference abstracts in German language**

1. Abstractband DOG 2022. *Ophthalmologie* **119** (Suppl 3), 157–344 (2022). <https://doi.org/10.1007/s00347-022-01723-2>
2. Abstractband DOG 2023. *Ophthalmologie* **120** (Suppl 2), 71–253 (2023). <https://doi.org/10.1007/s00347-023-01901-w>
3. Abstracts zum 112. DOG-Kongress. *Ophthalmologe* **111** (Suppl 1), 1–172 (2014). <https://doi.org/10.1007/s00347-014-3133-x>
4. Abstract-Band DOG 2014. *Ophthalmologe* **112** (Suppl 1), 1–180 (2015). <https://doi.org/10.1007/s00347-015-0116-5>

**Textbook Chapters**

1. Barrett, G.D. (2023). Cataract Surgery—Considerations When Planning Monovision. In: Shajari, M., Priglinger, S., Kohnen, T., Kreutzer, T.C., Mayer, W.J. (eds) Cataract and Lens Surgery. Springer, Cham. <https://doi.org/10.1007/978-3-031-05394-8_43>
2. Müller-Kassner, A., Shajari, M. (2023). Systematic Overview of Multifocal Intraocular Lenses. In: Shajari, M., Priglinger, S., Kohnen, T., Kreutzer, T.C., Mayer, W.J. (eds) Cataract and Lens Surgery. Springer, Cham. <https://doi.org/10.1007/978-3-031-05394-8_76>
3. Shajari, M., Alio, J.L. (2023). Indications for Multifocal and Extended Depth of Focus Intraocular Lenses. In: Shajari, M., Priglinger, S., Kohnen, T., Kreutzer, T.C., Mayer, W.J. (eds) Cataract and Lens Surgery. Springer, Cham. <https://doi.org/10.1007/978-3-031-05394-8_39>
4. Dirisamer, M. (2023). New Lenses. In: Tsui, E., Fung, S.S.M., Singh, R.B. (eds) Current Advances in Ocular Surgery. Current Practices in Ophthalmology. Springer, Singapore. https://doi.org/10.1007/978-981-99-1661-0_6
5. Arbisser, L.B. (2024). Intraocular Lens Selection in Cataract Surgery. In: Pardianto, G., Tassignon, MJ. (eds) Innovation in Cataract Surgery. Springer, Singapore. <https://doi.org/10.1007/978-981-97-5192-1_7>
6. Riaz, K.M. (2022). What’s on the Menu: An Overview of Currently Available IOLs and Relevant Optics. In: Riaz, K.M., Vicente, G.V., Wee, D. (eds) Optics for the New Millennium. Springer, Cham. <https://doi.org/10.1007/978-3-030-95251-8_27>
7. Chan, T.C.Y., Chow, S.S.W., Chang, J.S.M. (2021). Refractive Aim and Choice of Intraocular Lens. In: Liu, C., Shalaby Bardan, A. (eds) Cataract Surgery. Springer, Cham. <https://doi.org/10.1007/978-3-030-38234-6_6>
8. Coutts, S.J., Barsam, A. (2021). Market Forces, Premium Cataract Surgery and Managing the Unhappy Patient. In: Liu, C., Shalaby Bardan, A. (eds) Cataract Surgery. Springer, Cham. <https://doi.org/10.1007/978-3-030-38234-6_13>
9. Wong, A.C.M. (2022). Optics of Intraocular Lenses. In: Albert, D.M., Miller, J.W., Azar, D.T., Young, L.H. (eds) Albert and Jakobiec's Principles and Practice of Ophthalmology. Springer, Cham. <https://doi.org/10.1007/978-3-030-42634-7_239>
10. Alió del Barrio, J.L., Vega, A., Alió, J.L. (2022). Accommodative Intraocular Lenses. In: Albert, D.M., Miller, J.W., Azar, D.T., Young, L.H. (eds) Albert and Jakobiec's Principles and Practice of Ophthalmology. Springer, Cham. <https://doi.org/10.1007/978-3-030-42634-7_232>
11. Vasquez-Perez, A., Liu, C. (2021). What Do Cataract Patients Want?. In: Liu, C., Shalaby Bardan, A. (eds) Cataract Surgery. Springer, Cham. <https://doi.org/10.1007/978-3-030-38234-6_1>
12. Alió del Barrio, J.L., Vega, A., Alió, J.L. (2021). Accommodative Intraocular Lenses. In: Albert, D., Miller, J., Azar, D., Young, L.H. (eds) Albert and Jakobiec's Principles and Practice of Ophthalmology. Springer, Cham. <https://doi.org/10.1007/978-3-319-90495-5_232-1>
13. Piñero, D.P., Alió del Barrio, J.L., Camps, V.J. (2019). Extended Depth of Field Intraocular Lenses: Mini Well Ready Lens. In: Alió, J., Pikkel, J. (eds) Multifocal Intraocular Lenses. Essentials in Ophthalmology. Springer, Cham. <https://doi.org/10.1007/978-3-030-21282-7_27>
14. Cochener-Lamard, B. (2019). Multifocal Intraocular Lenses: The Johnson and Johnson Family of Lenses. In: Alió, J., Pikkel, J. (eds) Multifocal Intraocular Lenses. Essentials in Ophthalmology. Springer, Cham. <https://doi.org/10.1007/978-3-030-21282-7_21>
15. Wong, A.C.M. (2020). Optics of Intraocular Lenses. In: Albert, D., Miller, J., Azar, D., Young, L.H. (eds) Albert and Jakobiec's Principles and Practice of Ophthalmology. Springer, Cham. <https://doi.org/10.1007/978-3-319-90495-5_239-1>
16. Alió, J.L., Grzybowski, A., Kanclerz, P. (2019). Extended Depth-of-Field Intraocular Lenses. In: Alió, J., Pikkel, J. (eds) Multifocal Intraocular Lenses. Essentials in Ophthalmology. Springer, Cham. <https://doi.org/10.1007/978-3-030-21282-7_26>
17. Morris, C., Qazi, M.A., Pepose, J.S. (2019). Accommodative Intraocular Lenses: Crystalens. In: Alió, J., Pikkel, J. (eds) Multifocal Intraocular Lenses. Essentials in Ophthalmology. Springer, Cham. <https://doi.org/10.1007/978-3-030-21282-7_29>
18. Luft, N. (2024). Intraocular Lens Selection and Biometry for Cataract Surgery After SMILE. In: Sekundo, W., Wagner, F.M. (eds) Femtosecond Laser Assisted Lenticule Extraction. Springer, Cham. <https://doi.org/10.1007/978-3-031-60424-9_18>
19. Packer, M., Howard Fine, I., Hoffman, R.S., Burkhard Dick, H. (2006). Pseudoaccommodative and Accommodative IOLs. In: Kohnen, T., Koch, D.D. (eds) Cataract and Refractive Surgery. Essentials in Ophthalmology. Springer, Berlin, Heidelberg . <https://doi.org/10.1007/3-540-30796-6_10>
20. Schallhorn, J.M. (2023). Refractive Lens Exchange in High Myopia. In: Randleman, J.B. (eds) Refractive Surgery for High Myopia. Essentials in Ophthalmology. Springer, Cham. <https://doi.org/10.1007/978-3-031-40560-0_7>
21. Bostanci, B. (2023). Considerations in the Amblyopic Patient When Planning Cataract Surgery. In: Shajari, M., Priglinger, S., Kohnen, T., Kreutzer, T.C., Mayer, W.J. (eds) Cataract and Lens Surgery. Springer, Cham. <https://doi.org/10.1007/978-3-031-05394-8_42>
22. Breyer, D.R.H., Saad, A. (2024). Refractive Cataract Surgery with Advanced Technology Intraocular Lens (AT-IOL). In: Pardianto, G., Tassignon, MJ. (eds) Innovation in Cataract Surgery. Springer, Singapore. <https://doi.org/10.1007/978-981-97-5192-1_21>

**Review articles**

1. Hong, A.S.Y., Jin, E., Shen, L. *et al.* Monovision versus multifocality for presbyopia during primary phacoemulsification: systematic review and network meta-analysis. *Eye* (2024). <https://doi.org/10.1038/s41433-024-03454-x>
2. Song, T., Duan, X. Ocular dominance in cataract surgery: research status and progress. *Graefes Arch Clin Exp Ophthalmol* **262**, 33–41 (2024). <https://doi.org/10.1007/s00417-023-06216-9>
3. Cummings, A.B., Savini, G., Carones, F. *et al.* The Vivity IOL: the European Experience. *Curr Ophthalmol Rep* **10**, 11–18 (2022). <https://doi.org/10.1007/s40135-022-00283-7>
4. Shafer, B.M., Greenwood, M. Presbyopia Correction at the Time of Cataract Surgery. *Curr Ophthalmol Rep* **8**, 79–87 (2020). <https://doi.org/10.1007/s40135-020-00236-y>
5. Barnett, V., Barsam, A. Update on Laser Vision Correction Versus Intraocular Lens Options. *Curr Ophthalmol Rep* **8**, 104–110 (2020). <https://doi.org/10.1007/s40135-020-00242-0>
6. Khandelwal, S.S., Jun, J.J., Mak, S. et al. Effectiveness of multifocal and monofocal intraocular lenses for cataract surgery and lens replacement: a systematic review and meta-analysis. Graefes Arch Clin Exp Ophthalmol 257, 863–875 (2019). <https://doi.org/10.1007/s00417-018-04218-6>
7. Grzybowski, A., Kanclerz, P. & Muzyka-Woźniak, M. Methods for evaluating quality of life and vision in patients undergoing lens refractive surgery. *Graefes Arch Clin Exp Ophthalmol* **257**, 1091–1099 (2019). <https://doi.org/10.1007/s00417-019-04270-w>
8. Baldassare, R., Bedi, R. Symfony Extended Depth of Focus IOL: a Review of Reported Data. *Curr Ophthalmol Rep* **5**, 225–231 (2017). <https://doi.org/10.1007/s40135-017-0143-2>
9. Alió, J.L., Alió del Barrio, J.L. & Vega-Estrada, A. Accommodative intraocular lenses: where are we and where we are going. *Eye and Vis* **4**, 16 (2017). <https://doi.org/10.1186/s40662-017-0077-7>
10. Menapace, R., Findl, O., Kriechbaum, K. *et al.* Accommodating intraocular lenses: a critical review of present and future concepts. *Graefe's Arch Clin Exp Ophthalmol* **245**, 473–489 (2007). <https://doi.org/10.1007/s00417-006-0391-6>
11. Fernández, J., Molina-Martín, A., Rocha-de-Lossada, C. *et al.* Clinical outcomes of presbyopia correction with the latest techniques of presbyLASIK: a systematic review. *Eye* **37**, 587–596 (2023). <https://doi.org/10.1038/s41433-022-02175-3>
12. He, Y., Zhu, B., Li, B. *et al.* Stereopsis Following Implantation of Presbyopia-Correcting Intraocular Lenses: A Narrative Review. *Ophthalmol Ther* **13**, 2331–2341 (2024). https://doi.org/10.1007/s40123-024-01004-y
